# Supplementary material for: Factors Influencing the Acceptance of COVID-19 Vaccines in a Country with a High Vaccination Rate
Source: Vaccines (Basel). 2022 Apr 25;10(5):681. doi: 10.3390/vaccines10050681 (PMC9145438; doi:10.3390/vaccines10050681)
Supplement: Supplementary file 1 [file vaccines-10-00681-s001.zip › vaccines-1656288-supplementary.pdf]

Supplementary information

Supplementary Table S1. Summary of socio-demographic characteristics of respondents included in this study (N=744).

**Socio demographic characteristics**		
Variable		N (%)
Age	18-29	206 (27.7)
	30-59	503 (67.6)
	> 59	35 (4.7)
Gender	Women	484 (65.0)
Education*	Middle School	5 (0.7)
	High School	273 (36.7)
	Undergraduate	311 (41.8)
	Postgraduate	155 (20.8)
Location	Capital of Chile	433 (58.2)
Nationality	Chilean	725 (97.5)
Infected with COVID-19	Yes	94 (12.6)

*Last complete educational level

Supplementary Table S2. Milestones that occurred during the data collection period, according to the development of the pandemic and vaccination process in Chile.

DATE	EVENT
**May 21**	The highest incidence rate in Chile reaches 12.7% .
**May 25**	Vaccination begins in people younger than 30 years.
**May 27**	6% increase in new cases nationwide compared to the last seven days.
**May 31**	2020 Public Account of the Chilean Ministry of Health.
**June 2**	The highest incidence rate in Chile reaches 11.9%.
**June 3**	Mobility Pass permits are restricted.
**June 4**	Chile achieved 15,053,577 PCR examinations, which places our country in a leadership position in Latin America and the world. The positivity in the last 24 hours is 10%.
**June 7**	Chilean Ministry of Health reports that 75% of new COVID-19 cases haven't completed their vaccination schedule.
**June 10**	All of the communes in Santiago, the capital of Chile, are government mandated to go into lockdown.
**June 11**	Chilean Ministry of Health announces the imminent start of vaccinations in children between 17 and 12 years old with the Pfizer vaccine.
**June 13**	Elections of Regional Governors are held.
**June 15**	11 regions decreased their cases in the last seven days and 10 in the last 14 days.
**June 17**	An 11% decrease in cases in one week.
**June 19**	Conversation table between the Chilean Ministry of Health and different guilds and specialists to review health strategies.
**June 20**	Strategy proposed by the Medical College on "Epidemiological circuit-breaker" is rejected.
**June 21**	24 of 52 communes in Santiago, the capital of Chile, are released from lockdown.

Supplementary Table S3. Ranking of models to estimate the association of the outcome variables of the willingness to accept (a) a SARS-CoV-2 vaccine, (b) a third dose, (c) an annual vaccination, and (d) the vaccination of children, and explanatory variables of trust and perceptions.

	Willingness to accept:															
	(a) SARS-CoV-2 vaccine				(b) third dose				(c) annual vaccination				(d) vaccination of children			
Models	Omitted variables	k	AICc	Δ AICc	Omitted variables	k	AICc	Δ AICc	Omitted variables	k	AICc	Δ AICc	Omitted variables	k	AICc	Δ AICc
Core model	[20] to [23]	19	281.5	0.0	[20] to [23]	19	348.2	0.0	[20] to [23]	19	1287.3	0.0	[20] to [23]	21	1096.7	0.0
Candidate models	[20] to [22]	20	283.4	1.9	[20] to [22]	20	349.7	1.4	[20] to [22]	20	1288.3	1.0	[21], [23]	19	1096.8	0.1
									[20], [23]	21	1288.9	1.6	[23]	22	1097.5	0.8
									[16], [20]	21	1288.9	1.6	[22]	22	1097.9	1.3
									[20], [21]	21	1288.9	1.6	[16]	22	1097.9	1.3
									[20], [22]	21	1289.0	1.7	[21]	22	1098.5	1.8
									[21], [23]	21	1289.0	1.7	[20], [23]	21	1098.6	1.9
									[16]	22	1289.2	1.8				
Models with relatively little support	[20], [22]	21	285.5	4.0	[20], [21]	21	351.6	3.4	[22]	22	1289.7	2.4	[20] to [22]	20	1098.8	2.1
	[20], [21]	21	285.5	4.0	[16], [20]	21	351.6	3.4	[23]	22	1289.7	2.4	[20], [22]	21	1099.3	2.6
	[16], [20]	21	285.5	4.0	[20], [22]	21	351.8	3.5	[20]	22	1289.7	2.4	-	23	1099.4	2.7
	[21], [23]	21	285.6	4.1	[21], [23]	21	352.2	3.9	[21]	22	1289.9	2.6	[16], [20]	21	1099.9	3.3

[20],	2	28	4.2	[20],	2	35	4.0	-	2	129	3.1	[20],	2	110	3.5
[23]	1	5.7		[23]	1	2.3			3	0.4		[21]	1	0.1	
[22]	2	28	6.0	[21]	2	35	5.4					[20]	2	110	3.9
	2	7.5			2	3.6							2	0.5	
[21]	2	28	6.0	[20]	2	35	5.5								
	2	7.5			2	3.7									
[16]	2	28	6.1	[16]	2	35	5.5								
	2	7.6			2	3.7									
[20]	2	28	6.1	[22]	2	35	5.6								
	2	7.6			2	3.8									
[23]	2	28	6.2	[23]	2	35	6.1								
	2	7.7			2	4.3									
-	2	28	8.1	-	2	35	7.5								
	3	9.6			3	5.8									

Models varied in terms of the number of variables of trust and perceptions. k is the number of parameters in the model, including outcome and explanatory variables. AICc refers to Akaike's Information Criterion adjusted for a small sample size and Δ AICc is the difference in the AICc value of each model from the Core model. Models were grouped according to AICc value in the core model (smallest value), candidate models (Δ AICc <2), and models with relatively little support (Δ AICc>2). Explanatory variables: [1] trust in vaccines, [2] trust in scientists and medical professionals, [3] trust in politicians, [4] trust in religious leaders, [5] trust in relatives, [6] trust in social media, [7] trust in press, [8] perceived effectiveness of prevention practices, [9] perceived risk of infection, [10] preoccupation regarding side effects, [11] perceived comprehension of vaccines, [12] perceived prevention of severity of illness due to vaccines, [13] perceived relaxation of prevention practices thanks to vaccination, [14] perceived possibility of the vaccination stopping the pandemic, [15] perceived impact on quality of life, [16] COVID-19 infection in the family, [17] age, [18] gender, [19] schooling, [20] been sick with COVID-19, [21] region of residence, [22] acute COVID-19 infection of a family member, [23] nationality.

Supplementary Table S4. Associations of the willingness to accept a SARS-CoV-2 vaccination, third dose, annual vaccination, and to vaccinate children with variables of trust and perception among women (n = 484) and men individuals (n = 260).

		Willingness to receive a SARS-CoV-2 vaccination		Willingness to receive a third dose vaccination		Willingness to receive an annual vaccination		Willingness to vaccinate children	
		Women	Men	Women	Men	Women	Men	Women	Men
Explanatory variables		[1]	[2]	[3]	[4]	[5]	[6]	[7]	[8]
Trust in vaccines	[a]	4.8** (2.1 - 11.0)	5.8 (0.7 - 50.0)	4.0** (1.9 - 8.2)	3.4 (0.9 - 12.1)	2.4** (1.6 - 3.5)	1.8* (1.1 - 3.2)	2.1** (1.4 - 3.2)	1.8 (1.0 - 3.2)
Trust in scientists and medical professionals	[b]	2.2 (0.9 - 5.2)	46.1* (2.5 - 862.1)	1.9 (0.9 - 3.9)	4.2* (1.3 - 13.1)	2.0** (1.3 - 3.0)	2.5** (1.4 - 4.4)	2.0** (1.3 - 3.0)	3.8** (2.1 - 6.9)
Trust in politicians	[c]	3.1* (1.2 - 8.2)	0.6 (0.0 - 18.3)	2.4* (1.1 - 5.3)	1.0 (0.3 - 2.9)	1.2 (0.9 - 1.7)	1.2 (0.7 - 2.0)	1.3 (0.9 - 1.9)	1.1 (0.6 - 2.1)
Trust in religious leaders	[d]	0.9 (0.4 - 2.1)	2.0 (0.0 - 111.8)	0.8 (0.4 - 1.6)	0.4 (0.2 - 1.0)	0.8 (0.6 - 1.1)	0.7 (0.5 - 1.1)	0.7* (0.5 - 1.0)	0.6* (0.4 - 0.9)
Trust in relatives	[e]	1.2 (0.6 - 2.8)	8.0 (0.6 - 112.6)	1.7 (0.8 - 3.7)	1.3 (0.5 - 3.3)	1.1 (0.8 - 1.5)	1.1 (0.7 - 1.7)	1.0 (0.7 - 1.4)	1.8* (1.1 - 2.9)
Trust in social media	[f]	1.1 (0.4 - 2.7)	0.1 (0.0 - 1.3)	0.3** (0.1 - 0.7)	0.5 (0.2 - 1.3)	1.2 (0.8 - 1.8)	0.8 (0.5 - 1.3)	0.7 (0.4 - 1.0)	0.7 (0.4 - 1.2)
Trust in press	[g]	0.5 (0.2 - 1.4)	12.0 (0.4 - 343.8)	2.3 (1.0 - 5.6)	1.1 (0.4 - 3.3)	1.1 (0.7 - 1.6)	1.1 (0.7 - 1.8)	1.3 (0.9 - 2.0)	1.0 (0.6 - 1.8)
Perceived effectiveness of prevention practices	[h]	3.2* (1.1 - 9.5)	11.1 (0.9 - 133.3)	4.3** (1.7 - 11.3)	1.8 (0.6 - 5.1)	3.1** (1.8 - 5.1)	1.8* (1.0 - 3.2)	3.9** (2.3 - 6.7)	1.4 (0.7 - 2.5)
Perceived risk of infection	[i]	2.4* (1.1 - 5.1)	1.9 (0.3 - 13.4)	1.7 (0.9 - 3.1)	1.1 (0.5 - 2.5)	1.3 (0.9 - 1.8)	1.4 (0.9 - 2.2)	1.2 (0.9 - 1.7)	1.0 (0.6 - 1.6)
Preoccupation regarding side effects of vaccines	[j]	0.5** (0.3 - 0.8)	0.2* (0.0 - 0.9)	1.1 (0.7 - 1.8)	0.6 (0.3 - 1.2)	1.0 (0.8 - 1.2)	0.8 (0.6 - 1.1)	0.9 (0.7 - 1.1)	0.7* (0.5 - 0.9)
Perceived comprehension of vaccines	[k]	0.8 (0.4 - 1.7)	1.1 (0.1 - 8.5)	0.9 (0.4 - 1.9)	0.8 (0.3 - 2.4)	1.1 (0.8 - 1.7)	1.2 (0.7 - 1.9)	1.0 (0.7 - 1.5)	1.3 (0.8 - 2.3)
Perceived prevention of severity of illness due to vaccines	[l]	1.0	35.0**	1.0	1.0	1.0	1.0	0.8*	0.7*

		(0.6 - 1.5)	(2.7 - 462.4)	(0.7 - 1.5)	(0.6 - 1.7)	(0.8 - 1.2)	(0.8 - 1.3)	(0.7 - 1.0)	(0.6 - 1.0)
Perceived relaxation of prevention practices thanks to vaccination	[m]	1.3	3.5	0.9	0.6	0.7**	0.7	1.2	0.9
		(0.7 - 2.3)	(0.5 - 23.7)	(0.5 - 1.5)	(0.3 - 1.2)	(0.5 - 0.9)	(0.5 - 1.0)	(0.9 - 1.6)	(0.6 - 1.3)
Perceived possibility of the vaccination stopping the pandemic	[n]	1.0	14.6**	1.2	2.1*	1.4**	1.6**	1.2*	1.5**
		(0.6 - 1.6)	(1.9 - 111.7)	(0.8 - 1.8)	(1.2 - 3.9)	(1.2 - 1.7)	(1.2 - 2.1)	(1.0 - 1.5)	(1.1 - 2.0)
Perceived impact on quality of life	[o]	0.7	0.1	1.3	0.5	1.0	0.6*	0.9	0.7
		(0.3 - 1.3)	(0.0 - 1.5)	(0.7 - 2.4)	(0.2 - 1.1)	(0.7 - 1.4)	(0.4 - 1.0)	(0.6 - 1.3)	(0.4 - 1.0)
COVID-19 infection in family	[p]	0.4	31.7	0.7	2.2	1.1	1.0	0.7	1.0
		(0.2 - 1.1)	(0.4 - 2,465.2)	(0.3 - 1.5)	(0.5 - 8.5)	(0.7 - 1.7)	(0.6 - 1.9)	(0.5 - 1.2)	(0.5 - 2.0)
Age	[q]	1.1*	1.2*	1.0	1.0	1.0	1.0	1.0**	1.0**
		(1.0 - 1.1)	(1.0 - 1.4)	(0.9 - 1.0)	(1.0 - 1.1)	(1.0 - 1.0)	(1.0 - 1.0)	(1.0 - 1.1)	(1.0 - 1.1)
Schooling	[r]	1.0	0.5	0.9	1.0	0.9	1.0	0.9	0.8
		(0.7 - 1.4)	(0.2 - 1.1)	(0.7 - 1.2)	(0.7 - 1.5)	(0.8 - 1.0)	(0.8 - 1.2)	(0.8 - 1.1)	(0.7 - 1.0)
Multivariate model		Ordered logit	Ordered logit	Logit	Logit	Ordered logit	Ordered logit	Ordered logit	Ordered logit
Observations		484	260	484	260	484	260	484	260

Note = Columns [1], [2], [5], [6], [7], and [8] show results of ordered logit multivariate models. Columns [3] and [4] show logit model results. For all of the columns, cells show odds ratio coefficients and, in parenthesis, confidence intervals at 95%. For each outcome variable, Supplementary Table 4 shows the model with the best goodness of fit and parsimony compared with other candidate models, which was selected using Akaike Information Criterion (see Supplementary Table 3). * and ** refer to significant levels at 5% and 1%.

Supplementary Table S5. Associations of the willingness to accept a SARS-CoV-2 vaccination, third dose, annual vaccination, and the vaccination of children, with variables of trust and perception among young adults (<30 years old, n = 206) and adults (>29 and <60 years old, n = 503).

		Willingness to receive a SARS-CoV-2 vaccination		Willingness to receive a third dose vaccination		Willingness to receive an annual vaccination		Willingness to vaccinate children	
		Young adults	Adults	Young adults	Adults	Young adults	Adults	Young adults	Adults
Explanatory variables		[1]	[2]	[3]	[4]	[5]	[6]	[7]	[8]
Trust in vaccines	[a]	6.0**	3.4*	9.9**	2.2*	2.3**	1.9**	2.8**	1.5
		(1.6 - 21.7)	(1.1 - 10.0)	(2.4 - 41.6)	(1.0 - 4.9)	(1.4 - 3.9)	(1.3 - 2.9)	(1.6 - 4.8)	(1.0 - 2.4)
Trust in scientists and medical professionals	[b]	2.9	3.7*	1.2	3.9**	1.6	2.6**	1.1	3.9**
		(0.7 - 12.6)	(1.2 - 11.8)	(0.3 - 4.4)	(1.8 - 8.5)	(0.8 - 3.0)	(1.7 - 3.9)	(0.6 - 2.2)	(2.5 - 6.2)
Trust in politician	[c]	3.3	4.9*	1.5	2.1	1.2	1.2	2.1*	1.2
		(0.7 - 15.3)	(1.3 - 18.5)	(0.4 - 5.4)	(1.0 - 4.5)	(0.7 - 2.1)	(0.8 - 1.7)	(1.2 - 3.8)	(0.8 - 1.8)
Trust in religious leaders	[d]	1.8	0.7	0.5	0.5*	0.6*	0.8	0.4**	0.7
		(0.3 - 9.3)	(0.3 - 1.7)	(0.1 - 1.6)	(0.3 - 1.0)	(0.3 - 1.0)	(0.6 - 1.1)	(0.2 - 0.8)	(0.5 - 1.0)
Trust in relatives	[e]	0.4	3.9**	6.0*	1.2	1.1	1.1	1.1	1.3
		(0.1 - 1.4)	(1.5 - 9.9)	(1.3 - 28.7)	(0.6 - 2.3)	(0.6 - 2.0)	(0.8 - 1.6)	(0.6 - 2.0)	(0.9 - 1.9)
Trust in social media	[f]	3.6	0.6	0.2*	0.3**	1.1	0.9	0.7	0.7
		(0.7 - 17.0)	(0.2 - 1.8)	(0.1 - 1.0)	(0.2 - 0.7)	(0.6 - 2.1)	(0.7 - 1.3)	(0.4 - 1.3)	(0.5 - 1.0)
Trust in press	[g]	0.4	0.7	1.9	1.3	1.0	1.1	1.4	1.0
		(0.1 - 2.1)	(0.2 - 2.4)	(0.5 - 7.6)	(0.6 - 2.9)	(0.6 - 1.8)	(0.8 - 1.6)	(0.8 - 2.6)	(0.6 - 1.5)
Perceived effectiveness of prevention practices	[h]	2.9	2.3	6.5	2.1	4.7**	1.9**	2.8*	2.3**
		(0.4 - 21.8)	(0.9 - 5.9)	(0.8 - 53.5)	(1.0 - 4.2)	(1.9 - 11.3)	(1.2 - 2.9)	(1.2 - 6.9)	(1.4 - 3.6)
Perceived risk of infection	[i]	3.6	1.3	1.7	1.3	1.3	1.3	1.3	1.1
		(1.0 - 13.2)	(0.6 - 3.1)	(0.5 - 5.2)	(0.8 - 2.3)	(0.8 - 2.2)	(1.0 - 1.8)	(0.8 - 2.2)	(0.8 - 1.6)
Preoccupation regarding side effects of vaccines	[j]	0.3**	0.7	0.3*	0.9	0.9	0.9	0.8	0.7*
		(0.1 - 0.6)	(0.3 - 1.3)	(0.1 - 0.8)	(0.6 - 1.5)	(0.6 - 1.3)	(0.7 - 1.1)	(0.6 - 1.2)	(0.6 - 0.9)
Perceived comprehension of vaccines	[k]	1.5	0.4*	0.7	0.6	2.1*	0.9	1.6	0.9
		(0.3 - 6.5)	(0.2 - 1.0)	(0.2 - 2.3)	(0.3 - 1.2)	(1.1 - 3.8)	(0.6 - 1.2)	(0.9 - 3.0)	(0.6 - 1.4)
Perceived prevention of severity of illness due to vaccines	[l]	0.8	2.3**	1.8	0.9	0.9	1.0	0.7*	0.9
		(0.4 - 1.6)	(1.3 - 4.1)	(0.9 - 3.9)	(0.6 - 1.2)	(0.7 - 1.3)	(0.8 - 1.2)	(0.5 - 1.0)	(0.7 - 1.0)

Perceived relaxation of prevention practices thanks to vaccination	[m]	1.1	1.7	0.4	0.9	0.8	0.7**	0.7	1.2
		(0.4 - 3.0)	(0.9 - 3.4)	(0.1 - 1.3)	(0.6 - 1.4)	(0.5 - 1.2)	(0.6 - 0.9)	(0.4 - 1.1)	(0.9 - 1.5)
Perceived possibility of the vaccination stopping the pandemic	[n]	0.9	2.0*	2.4	1.3	1.4*	1.5**	1.3	1.4**
		(0.4 - 2.1)	(1.1 - 3.6)	(1.0 - 5.6)	(0.9 - 1.8)	(1.0 - 1.8)	(1.3 - 1.8)	(0.9 - 1.7)	(1.2 - 1.8)
Perceived impact on quality of life	[o]	0.6	0.4*	0.1*	1.1	0.8	0.8	0.7	0.8
		(0.2 - 2.1)	(0.2 - 0.8)	(0.0 - 0.7)	(0.6 - 1.9)	(0.5 - 1.4)	(0.6 - 1.1)	(0.4 - 1.3)	(0.6 - 1.1)
COVID-19 infection in family	[p]	0.2	0.7	0.2	0.9	1.2	0.9	0.4*	0.9
		(0.0 - 1.2)	(0.2 - 2.4)	(0.0 - 1.2)	(0.4 - 1.9)	(0.6 - 2.5)	(0.6 - 1.4)	(0.2 - 0.9)	(0.5 - 1.3)
Age	[q]	1.5*	1.0	1.0	1.0	0.9	1.0	1.1	1.1**
		(1.1 - 2.2)	(0.9 - 1.1)	(0.8 - 1.4)	(0.9 - 1.0)	(0.8 - 1.1)	(1.0 - 1.0)	(1.0 - 1.2)	(1.0 - 1.1)
Gender (Women=1)	[r]	1.8	1.6	3.6	1.2	1.6	1.1	1.3	1.5
		(0.2 - 13.3)	(0.5 - 5.4)	(0.5 - 26.1)	(0.5 - 2.6)	(0.7 - 3.4)	(0.7 - 1.7)	(0.6 - 3.0)	(1.0 - 2.5)
Schooling	[s]	0.8	0.9	0.9	1.0	1.0	0.9	1.0	0.8**
		(0.5 - 1.4)	(0.6 - 1.3)	(0.5 - 1.4)	(0.8 - 1.3)	(0.8 - 1.3)	(0.8 - 1.1)	(0.8 - 1.3)	(0.7 - 0.9)
Multivariate model		Ordered logit	Ordered logit	Logit	Logit	Ordered logit	Ordered logit	Ordered logit	Ordered logit
Observations		206	503	206	503	206	503	206	503

Notes

Note 1= Elderly people (>59 years old) were omitted from models because of the sample size (n=35).

Note 2= Columns [1], [2], [5], [6], [7], and [8] show the results of the ordered logit multivariate models. Columns [3] and [4] show logit model results. For all of the columns, cells show odds ratio coefficients and, in parenthesis, confidence intervals at 95%. For each outcome variable, Supplementary Table 5 shows the model with best goodness of fit and parsimony compared with other candidate models, which was selected using Akaike Information Criterion (see Supplementary Table 3). * and ** refer to significant levels at 5% and 1%.

Questionnaire

VACCINE ACCEPTANCE SURVEY
"VACCINE AGAINST SARS-COV-2 IN CHILE: UNDERSTANDING THE DETERMINANTS ASSOCIATED WITH THE POPULATION'S WILLINGNESS TO ACCEPT IT"

A1. How old are you? ___________

A2. What is your gender?
1. Men
2. Women
3. Other

A3. What is your nationality?
1. Chilean
2. Peruvian
3. Colombian
4. Venezuelan
5. Bolivian
6. Haitian
7. Argentinian
8. Dominican
9. Other Which? ___ **OPEN TEXT BOX ANCHORED, MAX 250 CHARACTERS**
   Does not know – Does not answer

A4. What Region do you live in?
1. I Region (Tarapacá)
2. II Region (Antofagasta)
3. III Region (Atacama)
4. IV Region (Coquimbo)
5. V Region (Valparaíso)
6. VI Region (Libertador Bernardo O'Higgins)
7. VII Region (Maule)
8. VIII Region (Biobío)
9. IX Region (La Araucanía)
10. X Region (Los Lagos)
11. XI Region (Aysén)
12. XII Region (Magallanes)
13. Metropolitan Region
14. XIV Region (Los Ríos)
15. XV Region (Arica y Parinacota)
16. XVI Region (Ñuble)

A5. ZONE

North	Center	South	RM
1	2	3	4

A6. PLEASE INSERT NSE CHILE

A7. What is your commune of residence?

A8. What is your educational level?
1. Incomplete primary school
2. Completed primary school
3. Incomplete secondary school
4. Completed secondary school
5. Incomplete technical studies
6. Completed technical studies
7. Incomplete undergraduate studies
8. Completed undergraduate studies
9. Postgraduate studies

A9. Do you have children under 16 years old?
1. Yes
2. No

A10. What choice best represents your current situation regarding the vaccine?
1. Already vaccinated with the 1st or 2nd dose
2. Waiting to get vaccinated
3. Have not decided if I will get vaccinated
4. Will not get vaccinated

QUESTIONS ABOUT COVID-19 SEVERITY

A11. Have you gotten sick with COVID-19?
1. No
2. Yes, asymptomatic
3. Yes, symptomatic, no hospitalization
4. Yes, hospitalized

A12. Has a member of your family (father, mother, siblings, kids) gotten sick with COVID-19?
1. No -> **SKIP TO A16**
2. Yes, asymptomatic
3. Yes, symptomatic, no hospitalization
4. Yes, hospitalized
5. Yes, died

A13. Did the person that got COVID-19 live with you?
1. Yes

2. No

A14. How likely do you think it is to contract and get sick with COVID-19?
1. Unlikely
2. Little likely
3. Likely
4. Highly likely

TRUST IN THE SOURCE OF VACCINE INFORMATION

A15. How much do you trust the information about COVID-19 vaccines provided by?

		No trust	Little trust	Some trust	High trust
1	Family	1	2	3	4
2	Friends	1	2	3	4
3	Religious leaders	1	2	3	4
4	Politicians	1	2	3	4
5	Ministry of Health	1	2	3	4
6	Medical professionals	1	2	3	4
7	Scientists	1	2	3	4
8	ISP (Instituto de Salud Pública) professionals	1	2	3	4
9	WHO (World Health Organization) professionals	1	2	3	4

A16. How much do you trust the information about COVID-19 vaccines provided by?

		No trust	Little trust	Some trust	High trust	Do not use that media
1	Facebook	1	2	3	4	5
2	Instagram	1	2	3	4	5
3	Tik Tok	1	2	3	4	5
4	Twitter	1	2	3	4	5
5	WhatsApp	1	2	3	4	5
6	Websites	1	2	3	4	5
7	National television	1	2	3	4	5
8	International television	1	2	3	4	5
9	National newspaper	1	2	3	4	5
10	International newspaper	1	2	3	4	5
11	Radio	1	2	3	4	5

TRUST IN THE VACCINE

A17. How worried do you feel about the possible side effects of COVID-19 vaccines?

1. Not worried
2. Little worried
3. Some worried
4. Highly worried

A18. When deciding whether to vaccinate or not against COVID-19, how relevant is it for you the time it took to develop a COVID-19 vaccine?

1. Not relevant
2. Little relevant
3. Relevant
4. Highly relevant

A19. According to what you know or have heard, how effective do you think the following measures are to prevent COVID-19?

		Not effective	Little effective	Some effective	Highly effective	Do not know
1	Vaccination	1	2	3	4	99
2	Quarantine	1	2	3	4	99
3	Use of mask	1	2	3	4	99
4	Hand-washing	1	2	3	4	99
5	Keeping physical distance	1	2	3	4	99
6	Avoiding social gatherings	1	2	3	4	99
7	Home isolation	1	2	3	4	99
8	Use of sanitary clinics	1	2	3	4	99

A20. How much information do you have about the following vaccines?

		No information	Little information	Enough information	A lot of information	Do not know
1	CoronaVac (China)	1	2	3	4	99
2	Pfizer (Germany/USA)	1	2	3	4	99
3	CanSino (China /Canada)	1	2	3	4	99
4	Oxford-AstraZeneca (UK/Sweden)	1	2	3	4	99
5	Sputnik V (Russia)	1	2	3	4	99
6	Johnson & Johnson (USA/Belgium)	1	2	3	4	99

A21. How much do you trust in the following vaccines?

		No trust	Little trust	Some trust	High trust	Do not know
1	CoronaVac (China)	1	2	3	4	99
2	Pfizer (Germany/USA)	1	2	3	4	99
3	CanSino (China /Canada)	1	2	3	4	99
4	Oxford-AstraZeneca (UK/Sweden)	1	2	3	4	99
5	Sputnik V (Russia)	1	2	3	4	99
6	Johnson & Johnson (USA/Bélgica)	1	2	3	4	99

A22. If you had to pay for a COVID-19 vaccine, would you get vaccinated?

1. Yes
2. No

99. Do not know

A23. In the hypothetical situation that you already had two doses of the COVID-19 vaccine and you were informed that a booster dose were necessary, would you get the booster dose?

1. Yes
2. No

99. Do not know

A24. If it were necessary to get vaccinated against COVID-19 every year, as is the case with the Influenza vaccine, how willing would you be to get vaccinated every year?

1. Not willing
2. Little willing
3. Some Willing
4. Highly willing

POST VACCINATION IMPACT

A25. Do you agree or disagree with the following sentences?

		Highly disagree	Disagree	Do not agree or disagree	Agree	Highly agree	Do not know
1	With a vaccination you can stop using a mask	1	2	3	4	5	99
2	With a vaccination the measures to prevent physical interactions with other people can be relaxed	1	2	3	4	5	99
3	A vaccine will prevent me from contracting COVID-19	1	2	3	4	5	99
4	A vaccine will only prevent me from getting severely ill	1	2	3	4	5	99

5	Vaccinations will stop the COVID-19 pandemic	1	2	3	4	5	99

A26. **FILTER: (A11=1)** If a COVID-19 vaccine is approved for children under 16 years old, would you vaccinate your children?
FILTER: (A11=2) (If you had children under 16 years old and a COVID-19 vaccine were approved for them), would you vaccinate your children?

1. Definitively no
2. Probably no
3. Maybe yes
4. Definitely yes

A27. In your experience, how much has your life been impacted because of the pandemic in the following aspects?

		Very negatively	Negatively	Not negatively, not positively	Positively	Very positively
1	Your wellness	1	2	3	4	5
2	The jobs of your household members	1	2	3	4	5
3	The coexistence with your household members	1	2	3	4	5
4	The education of your household members	1	2	3	4	5
5	The health of your household members	1	2	3	4	5
6	The income of your household members	1	2	3	4	5

THANK YOU
YOU HAVE FINISHED THE SURVEY
